# Study on the application of optical coherence microscopy in Hirschsprung's disease

**DOI:** 10.1038/s41598-023-28341-5

**Published:** 2023-02-06

**Authors:** Zhiwei Wu, Jialun Song, Xianxu Zeng, Zhenjie Cao, Xinxin Zhao, Peng Wang, Qian Ma, Huan Ma, Baojin Wang, Junpeng Du

**Affiliations:** 1 Department of Pediatric Surgery, The Third Affiliated Hospital of Zhengzhou University, Zhengzhou, China; 2 Department of Pathology, The Third Affiliated Hospital of Zhengzhou University, Zhengzhou, China; 3 Department of Gynecology, The Third Affiliated Hospital of Zhengzhou University, Zhengzhou, China; 4 Henan International Joint Laboratory of Ovarian Malignancies, Zhengzhou, China; 5 Zhengzhou Guangchao Medical Technology Co Ltd., Zhengzhou, 450052 China

**Keywords:** Medical research, Paediatric research, Diseases, Gastrointestinal diseases

## Abstract

To explore the clinical application value of optical coherence microscopy (OCM) in Hirschsprung’s disease. 109 HSCR patients were recuited in a Chinese hospital from January 2018 to July 2021. All the recruited patients underwent barium enema angiography preoperatively and the resected diseased intestinal tubes were evaluated intraoperatively. The OCM and the histopathological examination were performed successively on the surgical specimens, and the OCM images were compared with the relevant tissue sections to characterize different lesions. 10 non-HSCR fetal colorectal tissues at the same period were retained for OCM, the characteristics of which with and without HSCR under OCM imaging were analyzed. In the OCM images of in vitro tissue, it can be clearly observed that the scattering degree of HSCR narrow segment mucosal is high, glands and crypt structures are reduced or even atrophy, and the scattering degree of submucosal and intermuscular is low; In the dilated segment, the low scattering and high scattering are complex, and the muscle layer is obviously hypertrophy and structural disorder. Compared with the pathological findings, the OCM sensitivity, Kappa value, and AUC area reached 92.66%, 0.63, and 0.91, respectively. OCM can quickly and clearly display the structure of all layers of colorectal tissue, which is highly consistent with the corresponding histopathological examination results and has high sensitivity. which will provide a more reliable basis for OCM diagnosis of early HSCR, targeted biopsy and location of operative treatment, and has a certain potential for clinical application.

## Introduction

**Figure 1:**
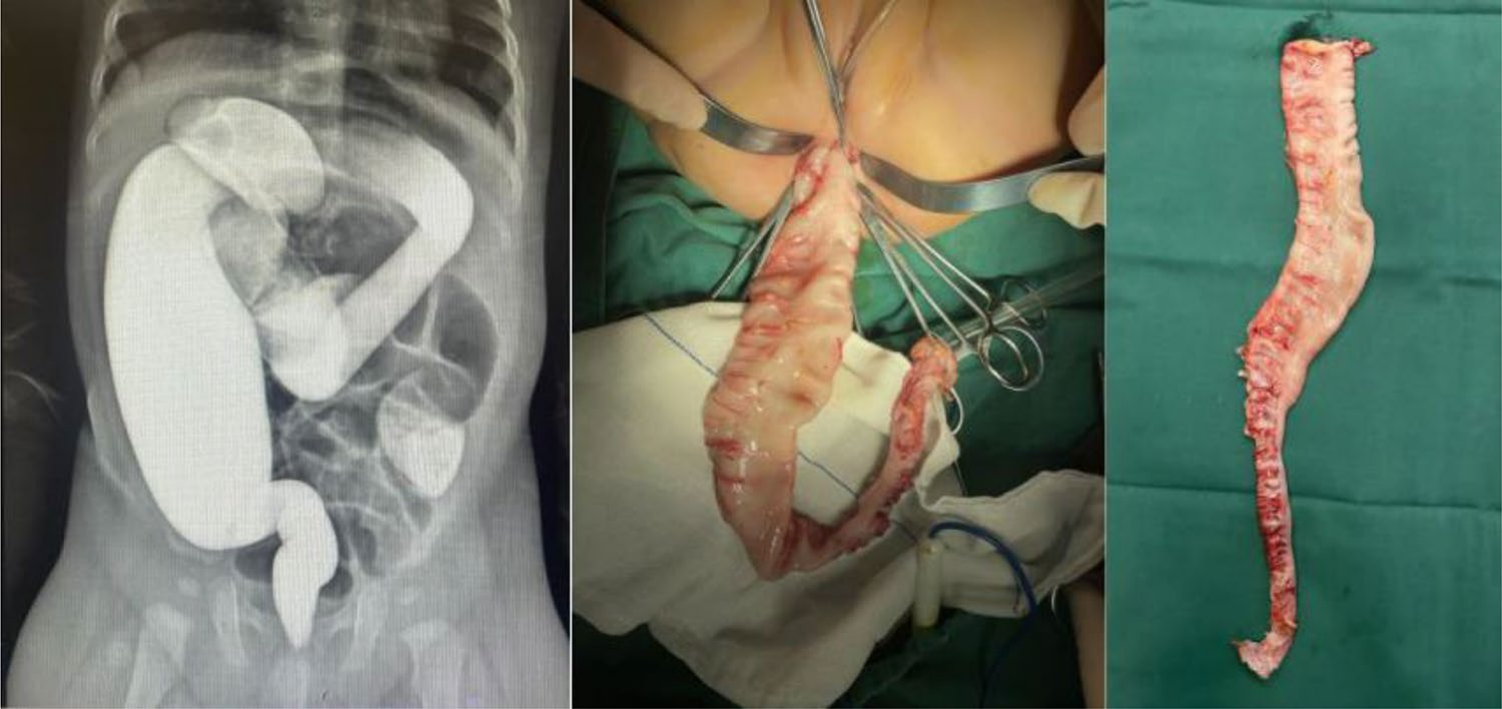
BE examination and surgical pathology specimens of the common type HSCR.

Hirschsprung's disease (HSCR), is a common abnormal developmental disease of intestinal neurons with an incidence of about 1 in 5000 and a prevalence four times higher in males than females. The pathological changes of HSCR are mainly caused by the lack of ganglion cells in the narrow muscular and submucosal plexus, whereas the thickening and number of exogenous nerve fibers are increased, in addition to cytoskeleton changes and smooth muscle thickening. According on the range of intestinal segments lacking ganglion cells, the clinical classification of the HSCR is included Short segment type, Common type, Long segment type, Total colonic type, Total intestine type. The typical general specimen can be divided into two parts: the proximal end of the diseased intestine is abnormally dilated, with a hypertrophic wall and slightly pale color; the distal dilated section is narrower, tends to be normal in size, and has no special appearance; and the transitional area between the two parts is mostly "funnel-shaped" (Fig. 1). The most common symptoms include neonatal intestinal obstruction, intractable constipation, and recurrent enterocolitis^1^. However, the early diagnosis of HSCR in small infants (age < 3 months) lacks the clinical manifestations of great diagnostic value, which makes ancillary diagnostic tests particularly important.

Barium enema (BE) is currently the most commonly used adjunctive diagnosis, with a sensitivity of 70% (64–76%) and a specificity of 83% (74–90%) for the diagnosis of HSCR^2^. The diagnostic accuracy of common and short-segment HSCR is higher, 80–90%, but if the transitional segment is located in the proximal sigmoid colon intestine, the accuracy becomes 50% to 60%^3^. The sensitivity of delayed film shooting (barium residue at 24 h) to diagnose HSCR is 85.7%, and the specificity is 17.6%^4^. The diagnostic process in pediatric HSCR (non-newborn) is relatively mature, but for the neonatal HSCR, it is difficult to make a correct diagnosis based on the imaging results due to the short time of fecal accumulation in the newborn, which makes the misdiagnosis rate significantly higher. Some studies have shown that the diagnosis of HSCR in preterm infants is significantly delayed compared with that in full-term infants because of the immature development of the enteric nervous system^5^, which also brings confusion for clinicians, so tissue biopsy is still the gold standard for diagnosis of HSCR. It consists of a preoperative rectal mucosa biopsy or postoperative histopathological examination to confirm HSCR. Rahman et al.^6^. Stated that this is invasive and impractical for the diagnosis and exclusion of HSCR in some suspected infants. Since the biopsy requires more tissue retrieval, there is still a possibility of missed examination, and it is an invasive examination that sometimes needs to be performed in the operating room, which is equivalent to additional surgery for children. The diagnosis method is costly and time-consuming, causing physical pain and psychological stress to patients and families, and also poses difficulties for clinicians in diagnosis and treatment.

Optical coherence tomography (OCT) and optical coherence microscopy (OCM) techniques are the frontiers and hotspots of the emerging discipline "biomedical photonics" formed by the intersection of optics, electronics, computer technology and other disciplines^7^. The imaging depth of OCT/OCM in most tissues is about 2–3 mm. However, its resolution is 10–100 times higher than that of ultrasound, with the advantages of non-invasive, high spatial resolution and 3D real-time imaging^8^, which can clearly display the structural characteristics and pathological changes of biological tissues and has shown great potential in the detection and clinical research of diseases such as cutaneous^9,10^, ophthalmic^11,12^, cardiovascular^13^, cervical diseases^14,15^, and gastrointestinal^16^. However, the use of OCT/OCM techniques for the study of human HSCR in-vivo or ex-vivo has not been explored so far. In this study, we analyzed the value of OCM in the clinical application of HSCR by scanning and imaging in vitro colorectal tissues of children with HSCR and non-HSCR fetuses with OCM technique.

## Patients

From January 2018 to July 2021, 129 patients who were diagnosed with HSCR in the Third Affiliated Hospital of Zhengzhou University were collected, and a total of 109 cases met the criteria after screening by inclusion and exclusion criteria (Table 1). Among them, 88 cases were male, 21 cases were female with male/female = 4.19:1; age ranged from 1 to 73 (12.09 ± 15.27) months; body weight ranged from 3.3 to 22.0 (8.74 ± 4.09) kg. 10 non-HSCR fetuses induced during the same period were retained, with a mean gestational age of 31.68 weeks (30–34).

**Table 1:** Demographics of the HSCR.

Sex	Example number	Age (month)	Weight (kg)	Common type (example)	Long segment type (case)	Short segment type (example)
Male	88	11.16 ± 14.89	8.59 ± 4.18	54	6	28
Female	21	15.98 ± 16.58	9.37 ± 3.72	17	0	4
Total	109	12.09 ± 15.27	8.74 ± 4.09	71	6	32

Inclusion criteria: (1) Children with HSCR confirmed by histopathologic results of rectal mucosal biopsy or surgical specimens according to C*linical treatment guidelines—The Pediatric Surgery Branch Book* (2021 Revised Edition); (2) All children underwent laparoscopic-assisted transanal macrocolectomy (3) Children who agreed to participate in the prospective study and signed informed consent.

Exclusion criteria: (1) preoperative barium enema angiography was not performed; (2) Qualified surgical specimens were not retained (including expansion and narrow segments of the intestine); (3) Surgical specimens were not subjected to OCM scanning and image processing; (4) Inclusion criteria were not followed and relevant data were missing.

This study was approved by the Medical Ethics Committee of the Third Affiliated Hospital of Zhengzhou University (approval number: 2022-097-01). All studies were conducted in accordance with the relevant guidelines or regulations. The patient's legal guardian had signed the informed consent (Supplementary Information).

## Methods

### BE

Before the surgery, X-ray and BE were performed to all the recruited patients. Ioversol was diluted to a concentration of 50–75% with saline and then examined using a Shimadzu gastrointestinal X-ray machine. 10–14 Fr catheter was placed through the anus, and diluted iodofol contrast was slowly injected into the rectum, and the injection volume was gradually increased under X-ray fluoroscopy to visualize the rectum, all of the colon and cecum. After 24 h of examination, the abdominal plain films were taken again in bilateral and supine positions; the retention of ioversol was understood. The imaging results were reviewed by two radiologists in our hospital and conclusions were drawn. The typical HSCR common types are shown in Fig. 1.

### OCM and image processing

**Figure 2:**
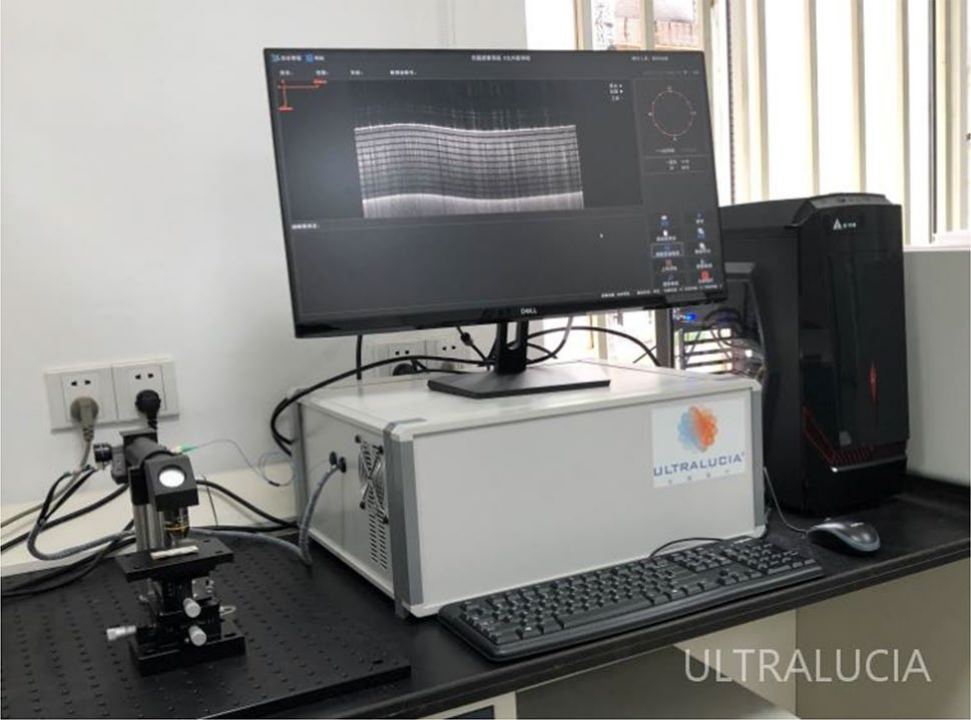
UL-D01-type optical coherence tomography system.

After intraoperative evaluation, some of the surgical specimens including the proximal dilated segment and the distal narrow segment of the diseased intestine were taken, OCM scanning and image processing were performed first. The OCM detection was performed using the UL-D01 type optical coherence tomography system (Zhengzhou Guangchao Medical Technology Co LTD) (Fig. 2). The steps were as follows: (1) Fully expand the intestinal tube mucosal surface, wipe the surface stains, and make a preliminary observation with the naked eye; (2) Scan the proximal dilated segment and distal narrow segment intestine, respectively with an orderly multi-dot scanning depth of 3 mm, 1.5 μm longitudinal resolution and 5 μm transverse resolution. After the OCM scans were completed, the specimens were fixed with 100 g/L neutral formaldehyde solution and sent to the pathology department for H&E staining; (3) The OCM images were identified by the trained researchers, to determine the tissue characteristics of the dilated and stenotic segments of the intestine by transverse and longitudinal comparisons and to record the abnormal sites. (4) To compare the OCM images with the corresponding histological images, firstly, we magnified by 10× the digital histological images of all biopsy sites obtained by scanning the H&E stained slice using a Mixotic scanner. In PowerPoint, the OCM images were appropriately scaled and adjusted to a 1:1 ratio in the axial and lateral directions for comparison with the corresponding histological sections. By carefully comparing the images, we obtained many well-matched OCM-H&E image pairs. Normal colorectal tissue from 10 induced non-HSCR fetuses were treated with the same method.

### Histopathological biopsy

**Figure 3:**
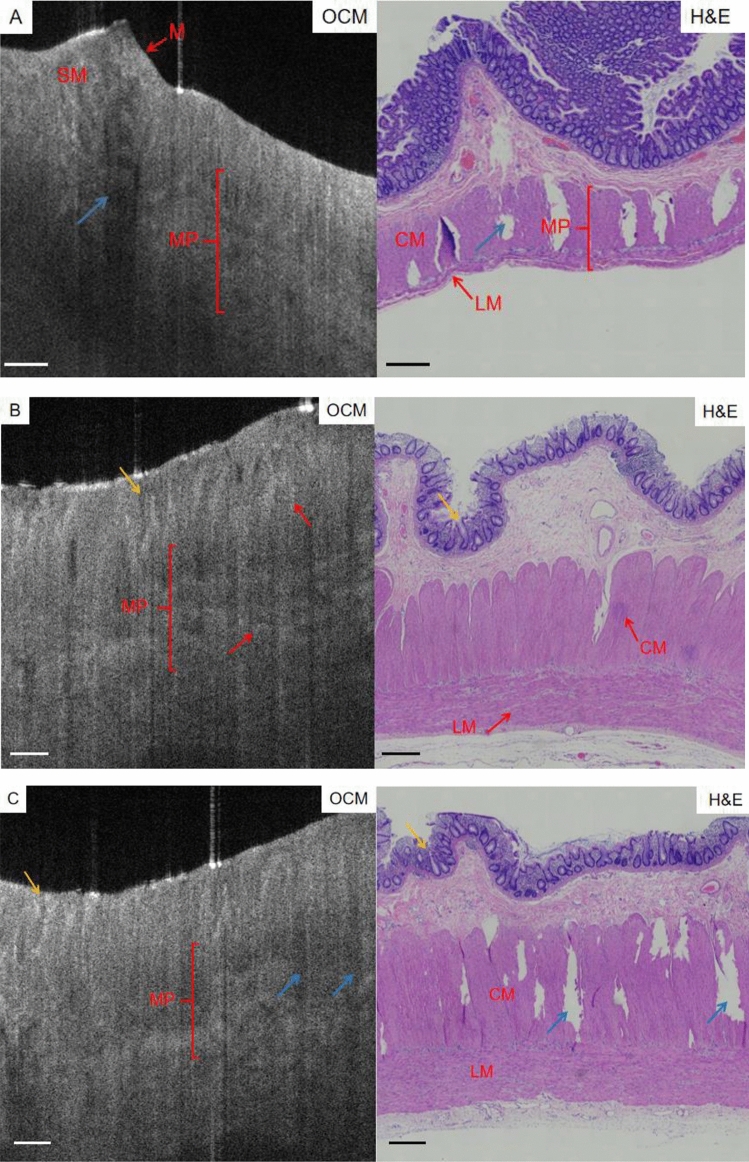
OCM images of HSCR dilated segments with H&E stained images. Scale bars 500 µm.

**Figure 4:**
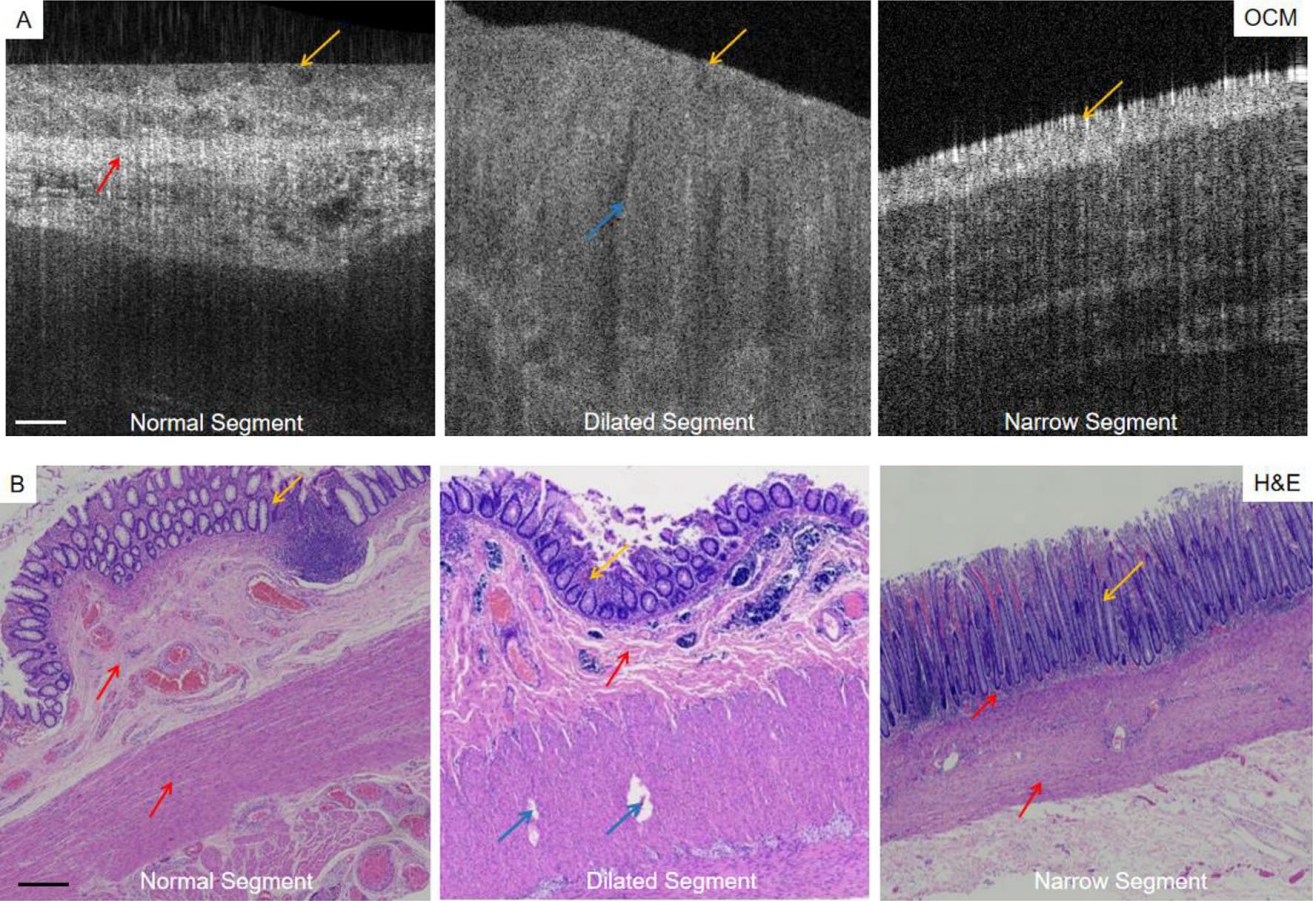
The OCM images of the colonic tissue without HSCR and with HSCR, together with the corresponding H&E stained images, Scale bars 500 µm.

Histopathological biopsies were taken from the proximal dilated segment and the distal narrow segment of the diseased intestine in children with HSCR, and the histopathological examination was performed after the completion of the OCM scan (Figs. 3, 4). H&E staining: paraffin-embedded, routinely sliced, double-blinded legal film and reviewed by two pathologists under the microscope. Immunohistochemical staining was performed if necessary to confirm the diagnosis. For normal colorectal tissue from 10 induced non-HSCR fetuses, the method was the same as above.

### Statistical analysis

Data were analyzed using SPSS 21.0 software to derive the sensitivity, and AUC values of BE and OCM for the diagnosis of HSCR. Pathological results were used as the "gold standard" for diagnosis and Kappa diagnostic agreement test was performed between BE and OCM results and histopathological results to observe the degree of agreement, and the greater the K value, the higher the anastomosis. The Chi-square test was used for group comparisons and *P* < 0.05 was considered statistically significant.

## Results

### OCM image features

Microstructures such as the serosal membrane, muscular layer, submucosa, some mucosal layers and crypts were clearly observed on the OCM images (M: Mucosa; SM: Submucosa; MP: Marina propria), which were successfully matched with the corresponding histological images (Figs. 3, 4).

Figure 3A–C shows the OCM images of the HSCR expansion segments with the corresponding H&E tissue sections. muscularis thickness increases, and there is a low scattering wide dark area (blue arrow) interspersed with higher scattering (muscle structure), this is the manifestation of muscularis structure disorder. The corresponding H&E tissue section showed the hypertrophy and structural disorder of the dilated intestinal tube muscle layer and showed the fracture performance (blue arrow).

Figure 4A,B shows the OCM images of the colon tissue without HSCR and with HSCR compared with the corresponding H&E staining images. OCM shows the scattering degree of mucosal layer: dilated segment < normal tissue < narrow segment, and the narrow segment mucosa is relatively bright (white), indicating a higher degree of light scattering; The structure of the layers without HSCR was relatively regular regular, and star dotted highlights areas were visible in submucosa and intermuscular. The muscle layer in HSCR has higher attenuation than that without HSCR, and the HSCR muscularis is heterogeneous and disorganized. Corresponding H&E stained images showed that the glands and crypt structures of colonic tissue with HSCR were reduced or even atrophied (yellow arrows), and no ganglion cells were seen. The mucosal muscle layer and intrinsic muscle layer of the dilated segment were thickened and hypertrophied, and structurally disorganized.

**Figure 5:**
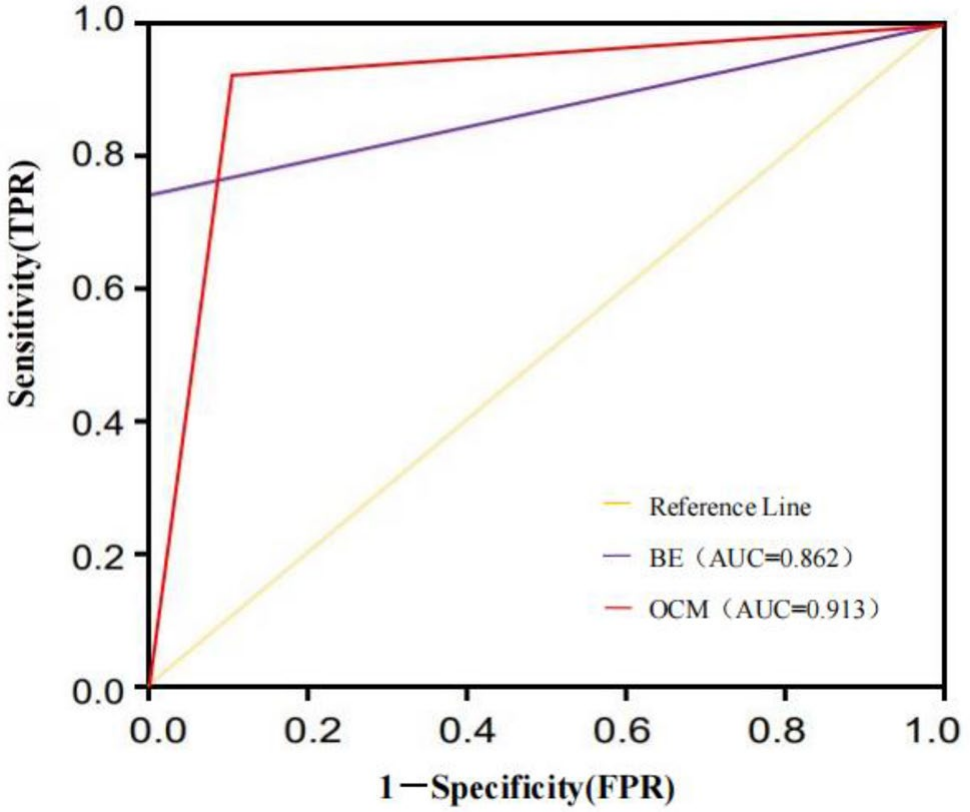
The ROC curves for the BE and OCM.

Among the 109 HSCR cases, 71 cases of common type, 32 cases of short segment type, and 6 cases of a long segment type. Using the results of the histopathological biopsy as the gold standard, when OCM was applied separately with BE, 101 cases had positive diagnostic results of OCM, with a sensitivity of 92.66% and AUC = 0.91; 79 cases had positive diagnostic results of BE, with a sensitivity of 72.48% and AUC = 0.86; the sensitivity and AUC of OCM examination were higher than those of BE examination (Fig. 5), and the difference in sensitivity was statistically significant (*χ*^2^ = 16.96, *P* < 0.001); the Kappa values of OCM and BE examination were 0.63, and 0.31, respectively.

There were 8 negative cases and 3 common types in BE examination, but their OCM scans all showed slight hypertrophy of the myenteric layer of the proximal rectum and disorganized structure compared with normal tissues, the postoperative pathological diagnosis results have all confirmed the short-segment type. Among the patients diagnosed with common type HSCR, the preoperative BE examination results were negative in 15 cases and short-segment type in 18 cases, however, the OCM scan could clearly show the abnormal manifestations of different intestinal segments, and then the initial diagnosis of HSCR was made, which was highly matched with the gold standard.

One of the aborted fetuses had colon dysplasia with a narrow distal intestinal canal and the OCM diagnosis was a false-positive result, which was confirmed by histopathological examination to be non-HSCR with the presence of ganglion and neural cells.

## Discussion

Optical coherence tomography (OCT) serves as a non-invasive, real-time, high-resolution in vivo imaging technology, while optical coherence microscopy (OCM) is a product of the combination of OCT and confocal microscopy, compared with OCT, OCM has a richer source of contrast and higher spatial resolution than OCT^17^. In 1997, OCT technology was first applied to the diagnosis and treatment of digestive tract diseases^18,19^. Studies related to the application of OCT technology in the human digestive tract have been reported in all sites such as esophagus, stomach, colon, bile duct and pancreatic^20–24^. At present, the research on this technology in the digestive system is progressing rapidly, especially through the combination of digestive endoscopy, puncture needle, fine needle, catheter and other instruments. According to the real-time OCT imaging features, it can accurately evaluate the mucosa, proper layer, mucosa, muscle layer and part of the submucosa, clearly determine the structural characteristics and properties of the disease, and has great potential in the early screening and diagnosis of gastrointestinal tumors; and shows great advantages in detecting the surgical margin of tumor lesions. Moreover, Hariri et al.^25^ have combined laparoscopy with OCT for the first time and successfully applied it intraoperatively, suggesting the feasibility of intraoperative laparoscopic OCT for real-time detection of surgical margins and lymph nodes to determine the appropriate extent of bowel resection and lymph node dissection. These studies fully demonstrated the advantages of OCT technology in disease screening, diagnosis, and surgical resection scope.

At present, the clinical methods for HSCR diagnostic examination has commonly used, including BE, ultrasound, anorectal pressure measurement, rectal mucosa biopsy, etc. While the difficulty in confirming the diagnosis preoperatively still exists, especially in newborns, when the ganglion cells are immature, and the complexity of histological manifestations often leads to the difficulty in judgment^26^. BE examination has some false positives and false negatives (about 20%), the diagnosis cannot be affirmed or excluded, and only helps provide the approximate location of the migrated segment for the reference of choosing the surgical access (transanal or laparoscopic aid)^27^. Especially in the neonatal period, contrast reagent causes pseudo-expansion of the narrow segment, easily misdiagnosis or miss^28^, this was also confirmed in this study. Confirming the diagnosis of HSCR still depends on rectal tissue biopsy (rectal mucosa or the whole layer). However, rectal mucosal biopsy has some disadvantages such as blindness, difficulty in precise localization of the lesion tissue, thickness and number of specimens in different cases, and the possibility of false negatives and inability to determine the extent of lesions in neonates. The results of this study show that OCM has a certain potential for clinical application in rapidly assisting HSCR diagnosis, targeted biopsy and location of operative treatment.

Westphal et al.^29^ obtained real-time endoscopic OCT images of fresh specimens of ileum, colon and rectum in a clinical trial, where the structures of mucosal and submucosal, glands, vessels, small pits, villi and crypts were observed. The results concluded that images provided by real-time endoscopic OCT were correlated with the tissue structure of gastrointestinal mucosa and submucosa. The visual stratified structure of the intestinal wall obtained by OCT/OCM scanning of human colon tissue was also confirmed in this study. Xiong et al.^30^ used OCT for the first time in SD rats to examine colon tissue without HSCR and with HSCR treatment for 3 and 6 weeks by establishing the HSCR Sprague–Dawley (SD) rat model and recording the change of colon tissue attenuation coefficient and myometrial thickness difference. The study initially demonstrated the feasibility of OCT for HSCR imaging, showing OCT in HSCR suspicious tissue in vivo diagnosis, and positioning targeted biopsy and invasive surgical treatment.

This study differed from the Xiong^30^, it exploits the principle of different reflectance intensities of different tissue structures and the high-resolution feature of OCM to detect human in vitro HSCR tissues for the first time, and We preliminarily summarize the OCM image features of human HSCR tissues and verify the feasibility of OCM for human HSCR imaging. In the OCM images, there are significant differences between the normal colon tissue and the HSCR, star punctate highlight area can be seen in the submucosa and intermuscle of normal colorectal rectum, which is considered as a manifestation of ganglion cell enrichment. The muscle layer in HSCR has higher attenuation than the muscle layer without HSCR, which may be induced by hypertrophy, altered cytoskeleton, vacuolization of muscle tissues and decreased myofilaments for clusters of myocytes^30^.

Our study has several deficiencies: (1) No criteria for determination of muscularis thickness are developed. (2) Sample selection is not comprehensive. First, the clinical type of HSCR is incomplete; Second, no other colon lesion cases are selected for control studies; Third, further investigations are necessary in order to evaluate the specificity of OCM in HSCR diagnosis. (3) In this study, the detection of ex-vivo specimens by OCM dose not reflect the advantages of real-time OCM in-vivo detection. In the future, OCM and colonoscopy can be combined to further investigate HSCR in-vivo. In summary, it requires future multicenter studies with large samples to develop appropriate HSCR diagnostic criteria by OCM.

## Conclusion

Our results suggest that OCM can identify HSCR-associated feature changes, which provides a new idea for the clinical auxiliary diagnosis of HSCR, provides a more reliable basis for targeted biopsy and location of operative treatment, and has a certain potential for clinical application.

## Supplementary Information

Supplementary Information.

## Data Availability

The datasets used and/or analyzed during the current study are available from the corresponding author on reasonable request.
