# Supplementary figures and images for: Study on the application of optical coherence microscopy in Hirschsprung's disease
Source: Sci Rep. 2023 Feb 6;13:2083. doi: 10.1038/s41598-023-28341-5 (PMC9902478; doi:10.1038/s41598-023-28341-5)

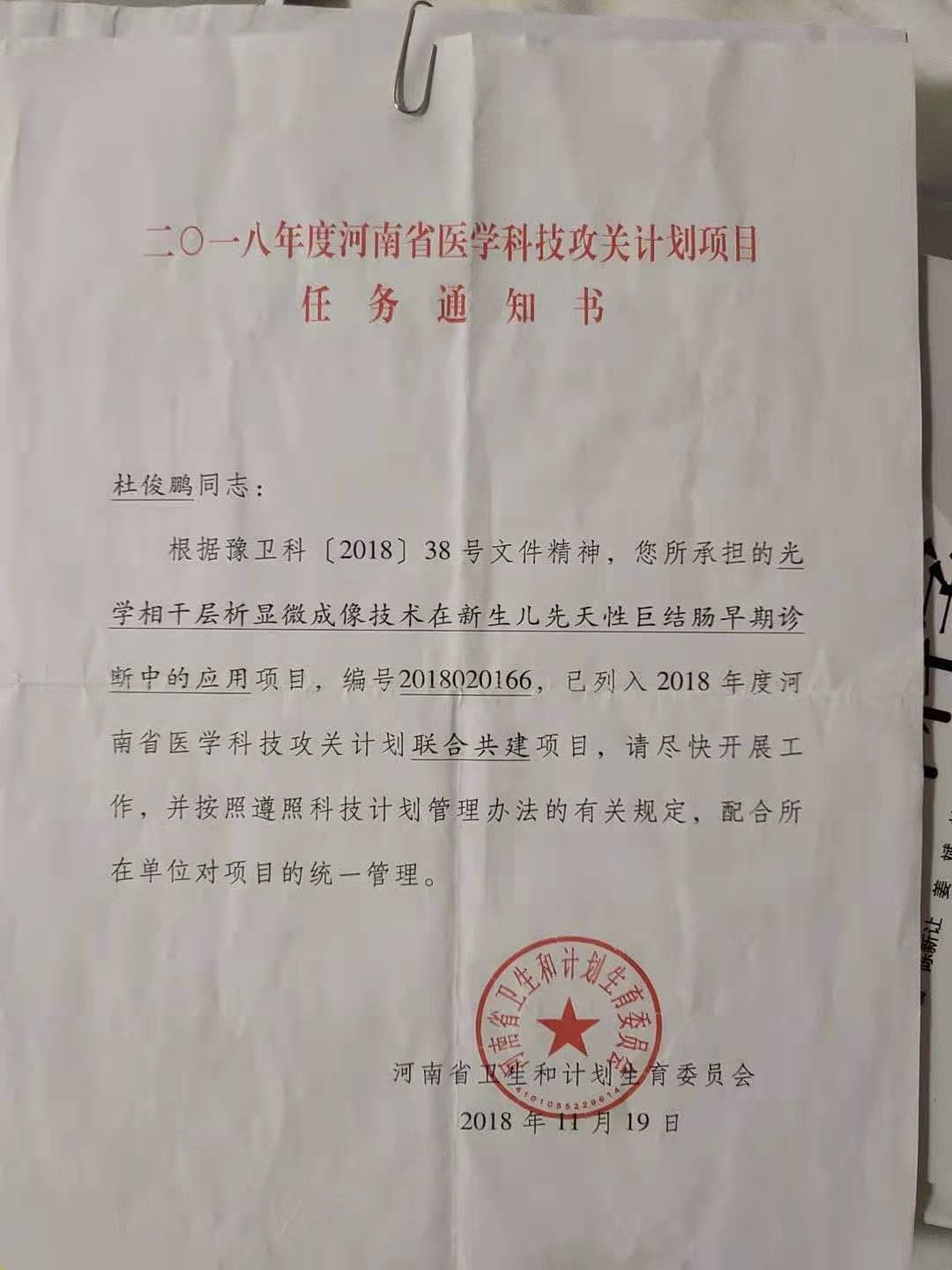

Supplement: Supplementary file 1 — Supplementary Information. [file 41598_2023_28341_MOESM1_ESM.jpg]
